# 629. Malaria Screening Turnaround Time: Comparative Multicenter Analysis of Negative and Positive Results and Impacts of Rapid Diagnostic Test Implementation and Laboratory Centralization

**DOI:** 10.1093/ofid/ofad500.695

**Published:** 2023-11-27

**Authors:** Louiselle LeBlanc, Alexie Rosa, Didier Martigny, Anne-Audrey Lapointe, Antoine Ricard-Lacombe, Mandy Malick, Simon Lévesque, Philippe Martin, Émile Provost, Stéphanie Bélanger, Christine Arsenault

**Affiliations:** Université de Sherbrooke, Sherbrooke, Quebec, Canada; Université de Sherbrooke/CIUSSSE-CHUS, Sherbrooke, Quebec, Canada; Université de Sherbrooke/CIUSSSE-CHUS, Sherbrooke, Quebec, Canada; Université de Sherbrooke/CIUSSSE-CHUS, Sherbrooke, Quebec, Canada; Université de Sherbrooke/CIUSSSE-CHUS, Sherbrooke, Quebec, Canada; Université de Sherbrooke, Sherbrooke, Quebec, Canada; Université de Sherbrooke, Sherbrooke, Quebec, Canada; Universite de Sherbrooke, Sherbrooke, QC, Canada; Université de Sherbrooke, Sherbrooke, Quebec, Canada; Université de Sherbrooke, Sherbrooke, Quebec, Canada; CIUSSS NIM - HSCM, Montreal, Quebec, Canada

## Abstract

**Background:**

Screening tests for malaria diagnosis should be reported rapidly because of its potential for rapid progression to severe disease and relatively high mortality. Guidelines recommend a high level of expertise for microscopy diagnosis and a laboratory test result turnaround time (TAT) ≤ 2 hours. Meeting these requirements can be challenging in the era of global laboratory centralization.

**Methods:**

Multicenter retrospective cohort study comparing negative (2012-2021) and positive (2014-2023) malaria screening results within and between 2 central laboratories/tertiary academic centers in Quebec, Canada, in addition to data from one center’s primary care affiliated sites.

**Results:**

1296 negative malaria tests were analyzed for complete analytical cycle TAT: Center A n=225 (central lab n=100; affiliated sites n=125), and Center B n=1120. After rapid diagnostic test (RDT) implementation, all sites have significantly reduced delays and ≤ 2h TAT compliance is > 90% for central labs (median: A 38 min, B 53min). But affiliated sites’ compliance is low at 19.6% (median 6h12) with delays mostly attributable during transit between labs (median TAT from central lab sample intake is 25 min). Comparison of positive malaria result TATs to negative result TATs for both server lab centers will be presented, with a trend to longer delays to smear results for negative RDTs (median 4h13) vs positive RDTs (3h33). Detailed results from central lab – affiliated sites model will be presented with several practical recommendations. In preliminary results, no cases were diagnosed solely with thick smear, suggesting little added sensitivity in our center when used in addition to RDT + thin smear.

**Conclusion:**

Quality and timely malaria diagnostic services are crucial for urgent clinical decisions given the potential for high morbidity and mortality. TATs are necessary lab quality management indicators during laboratory centralization and data for each component of malaria testing identifies weak links in the diagnostic chain. Such studies provide opportunities for quality and efficiency improvement, and for restructuring testing algorithms accordingly.

**Disclosures:**

**All Authors**: No reported disclosures

